# Analyzing the impact of surgical technique on intraoperative adverse events in laparoscopic Roux-en-Y gastric bypass surgery by video-based assessment

**DOI:** 10.1007/s00464-025-11557-z

**Published:** 2025-01-31

**Authors:** Joël L. Lavanchy, Deepak Alapatt, Luca Sestini, Marko Kraljević, Philipp C. Nett, Didier Mutter, Beat P. Müller-Stich, Nicolas Padoy

**Affiliations:** 1 University Digestive Health Care Center – Clarunis, PO box, 4002 Basel, Switzerland; 2 Department of Biomedical Engineering, University of Basel, Allschwil, Switzerland; 3 IHU Strasbourg, Strasbourg, France; 4 CNRS, INSERM, ICube, UMR7357, University of Strasbourg, Strasbourg, France; 5 Politecnico Di Milano, Milan, Italy; 6 Department of Visceral Surgery and Medicine, Inselspital, Bern University Hospital, University of Bern, Bern, Switzerland; 7 University Hospital of Strasbourg, Strasbourg, France

**Keywords:** Intraoperative adverse events, Postoperative complications, Surgical phases, Surgical steps, Workflow analysis, Surgical data science

## Abstract

**Background:**

Despite high-level evidence that variations of surgical technique in laparoscopic Roux-en-Y gastric bypass (LRYGB) are correlated with postoperative outcomes and might be linked to intraoperative adverse events (iAEs), there are a paucity of studies analyzing iAEs in depth. The impact of surgical technique on the temporal occurrence of iAEs regarding phases and steps of LRYGB has not been studied so far. The objective of this study was to analyze the impact of variance in surgical technique on temporal occurrence, frequency, and type of iAEs in a multicentric dataset of LRYGB videos.

**Methods:**

MultiBypass140, a video dataset containing 70 LRYGB surgeries each from Strasbourg University Hospital (StrasBypass70) and Bern University Hospital (BernBypass70) was annotated with surgical phases, iAE type, and grade. The cumulative severity of iAEs per procedure was measured using the SEVERE score and correlated with procedure duration.

**Results:**

Surgical technique significantly differed between StrasBypass70 and BernBypass70 (omentum division: 94% vs. 36%, *p* < 0.01; closure of mesenteric defects: 100% vs. 21%, *p* < 0.01). In MultiBypass140, a total of 797 iAEs were analyzed. The most iAE-prone phases were gastric pouch creation, gastrojejunal, and jejunojejunal anastomosis creation containing 77% (616/797) of all iAEs. StrasBypass70 showed significantly more iAEs in the omentum division (23 vs. 5, *p* < 0.01), Petersen space closure (13 vs. 1, *p* < 0.01), and mesenteric defect closure phases (34 vs. 1, *p* < 0.01) compared to BernBypass70. In both centers, SEVERE score was correlated with procedure duration. In BernBypass70, insufficient closure of anastomosis was significantly more frequent in patients with postoperative complications (0.2 ± 0.6 vs. 0.0 ± 0.1, *p* < 0.01).

**Conclusion:**

Variations of the LRYGB technique between centers influence the temporal occurrence and frequency of iAEs. The frequency and severity of iAEs are correlated with procedure duration.

**Supplementary Information:**

The online version contains supplementary material available at 10.1007/s00464-025-11557-z.

Currently, surgery is the most effective treatment for obesity and metabolic disorders [1, 2]. Worldwide, over 500,000 bariatric interventions are performed annually, of which the most frequent is laparoscopic Roux-en-Y gastric bypass (LRYGB) [3, 4]. The surgical technique of LRYGB is highly standardized and postoperative complications are low [5, 6]. Nevertheless, variations of LRYGB techniques are discussed to avoid intraoperative adverse events (iAEs) potentially translating into postoperative complications.

Division of the greater omentum, for example, was proposed to achieve tension-free antecolic gastrojejunostomy, as tension on the antecolic gastrojejunostomy can lead to postoperative small bowel obstruction. A recent registry-based cohort study from Sweden, including more than 40,500 patients undergoing LRYGB, showed that routine division of the greater omentum reduces postoperative small bowel obstruction. However, the reduced small bowel obstruction rate comes at the risk of increased iAEs like bleeding and small bowel injury [7].

Another well-discussed variation of surgical technique is the routine closure of mesenteric defects to prevent internal hernia. A randomized controlled trial from Sweden including 12 centers and 2,500 patients showed that routine closure of mesenteric defect reduces the risk of small bowel obstruction at 3-year follow-up. However, the closure of mesenteric defects increased the risk for postoperative complications due to kinking of the jejunojejunostomy [8].

A retrospective analysis of over 26,000 LRYGB patients from the Scandinavian Obesity Surgery Registry revealed iAEs as strong risk factors for serious postoperative complications. The risk of leakage and deep/organ space surgical site infection were threefold increased, and the risk of bleeding doubled in patients with iAEs [9]. An institutional review of the ACS-NSQIP database containing over 9,000 abdominal surgery patients demonstrated an independent association of iAEs with deep/organ space surgical site infection, sepsis, and 30-day mortality [10]. Further, iAEs are independently associated with the procedure’s complexity [11]. Despite high-level evidence that postoperative complications are related to variations in surgical technique and might be linked to iAEs [9–11], there are a paucity of studies analyzing iAEs in conjunction with technique. Video-based assessment is a promising tool to analyze the understudied intraoperative phase of surgeries [12]. A review of intraoperative videos allows experts to identify iAEs and analyze them in the context of the surgical workflow. A single-center study from Canada used a video-based assessment of 120 LRYGB procedures to develop and evaluate a score measuring the severity of iAEs [13]. However, iAEs were only assessed on a procedure level. During which part of the procedure iAEs occur was not studied. Therefore, a more granular study of iAE occurrence in specific stages of different procedures is needed. The implications thereof could shed light on the association of iAEs with the surgical technique.

This study was designed to improve the understanding of iAEs and to propose strategies to mitigate them. It analyzes the impact of surgical technique on the frequency, type, and grade of iAEs in relation to the surgical phases and steps in a multicentric dataset of 140 LRYGB videos.

## Material and methods

### Dataset and annotations

MultiBypass140, a multicentric dataset of 140 LRYGB videos consisting of 70 videos from Strasbourg University Hospital, France (referred to as StrasBypass70) and 70 videos from Inselspital, Bern University Hospital, Switzerland (referred to as BernBypass70), was used in this observational study [14]. Patients undergoing LRYGB surgery in one of both hospitals with complete video recordings of the procedure were retrospectively included into MultiBypass140. Surgical phases and steps of the procedures in the dataset were annotated by two surgeons with over ten years of clinical experience using an ontology of 12 phases, further divided into 46 steps that were validated for multicentric use (see Fig. S1, Supplemental Digital Content) [15]. The interobserver reliability of the used ontology was almost perfect (Cohen’s kappa 0.96 ± 0.04 and 0.81 ± 0.10 for phases and steps, respectively) [15]. iAEs within the dataset were annotated by a single surgeon using the SEVerity of intraoperative Events and REctification (SEVERE) index [13]. The surgeon annotator was trained based on the SEVERE index manual version 7. The SEVERE index contains 5 different types of iAEs (bleeding, thermal injury, mechanical injury, ischemic injury, and insufficient closure of anastomosis) and up to 5 severity grades per iAE type (Table 1). Rectification of iAE is assessed in a binary fashion (completely rectified vs. incompletely rectified). IAEs were considered completely rectified if the rectification process was satisfactory or the corresponding injury did not require rectification. The surgeon annotator watched the full MultiBypass140 video dataset and annotated the type, grade, and temporal duration of iAEs using the MOSaiC video annotation platform [16].

**Table 1:** Comparison of iAE classification systems and grades

	Severe [13]					ClassIntra [27]	EAES [29]
Grade	Bleeding	Thermal injury	Mechanical injury	Ischemic injury	Insufficient closure of anastomosis		
1	Very low amount of blood lost	Superficial penetration to “less vital” tissue	Superficial penetration to “less vital” tissue, needle poke to tissue	–	–	Any deviation from the ideal intraoperative course, without the need for any additional treatment or intervention, patient with no or mild symptoms	Minor error, no damage, or corrective action required
2	Low amount of blood lost	Deep penetration to “less vital” tissue or any organ/tissue subjected to planned resection	Full-thickness injury	–	–	Any deviation from the ideal intraoperative course, with the need for any additional minor treatment or intervention, patient with moderate symptoms, not life-threatening, and not leading to permanent disability	Minor consequential error requiring corrective action but no change in postoperative care
3	Intermediate amount of blood lost	Superficial penetration to “vital” tissue	Superficial penetration to “vital” tissue	Sign of ischemia with indeterminate nature	–	Any deviation from the ideal intraoperative course, with the need for any additional moderate treatment or intervention, patient with severe symptoms, potentially life-threatening or potentially leading to permanent disability	Consequential error requiring major corrective action and/or change in postoperative pathway
4	High amount of blood lost	Deep penetration to “vital” tissue to the level of muscularis/parenchyma	Deep penetration to “vital” tissue	–	–	Any deviation from the ideal intraoperative course, with the need for any additional major and urgent treatment or intervention, patient with life-threatening symptoms or leading to permanent disability	Life-threatening complication that requires major or immediate corrective action which led to a significant alteration of postoperative pathway
5	Very high amount of blood lost	Through and through injury to hollow organ or deeper parenchymal injury to solid organ	Through and through injury to “vital” tissue	Sign of permanent tissue ischemia	All insufficient closure of anastomosis	Any deviation from the ideal intraoperative course with intraoperative death of the patient	Major consequential error resulting in death

The SEVERE index was developed by analyzing 120 videos of LRYGB surgeries and showed excellent interobserver reliability (intraclass correlation coefficient 0.87, 95% CI 0.77–0.92) [13]. Based on single iAEs, a cumulative SEVERE score was calculated per procedure using the original SEVERE index item weights [13]. Major events were defined as 90th percentile contributing SEVERE score among the observed events in MultiBypass140. This corresponds to a SEVERE score ≥ 2.7. BernBypass70 included 3 attending surgeons after completion of the LRYGB learning curve. For BernBypass70, postoperative complications until 30 days postoperatively were recorded and graded using the Clavien–Dindo classification [17]. Major complications refer to Clavien–Dindo grades ≥ IIIa, which correspond to re-interventions and/or organ failure necessitating intensive care unit treatment. As StrasBypass70 is an anonymous dataset, no surgeon and patient data were available.

### Statistical analysis

Statistical analyses were performed using the SciPy.Stats, Statsmodel, and Scikit-learn libraries for Python. Categorical data are presented as number and frequency (%), and continuous data as mean ± standard deviation (SD). The Fisher exact test was used to compare iAE frequencies between centers. Normality of distribution was assessed using the Shapiro–Wilk test. The Mann–Whitney *U* test was used to compare average frequencies of minor and major events. The Pearson correlation coefficient was used to test the correlation of SEVERE score and procedure duration. A *p* value ≤ 0.05 was considered statistically significant.

This study is reported in accordance with the STROBE statement [18].

### Ethical approval

As the videos from Strasbourg University Hospital are anonymous, no institutional review board (IRB) approval was necessary. The use of videos from Inselspital, Bern University Hospital was approved by the IRB (Ethics Committee of the Canton of Bern 2021-01666) and the need to obtain informed consent was waived. All potentially privacy revealing scenes of MultiBypass140 were deidentified using OoBNet, a publicly available deep learning model for endoscopic video deidentification [19].

## Results

**Fig. 1:**
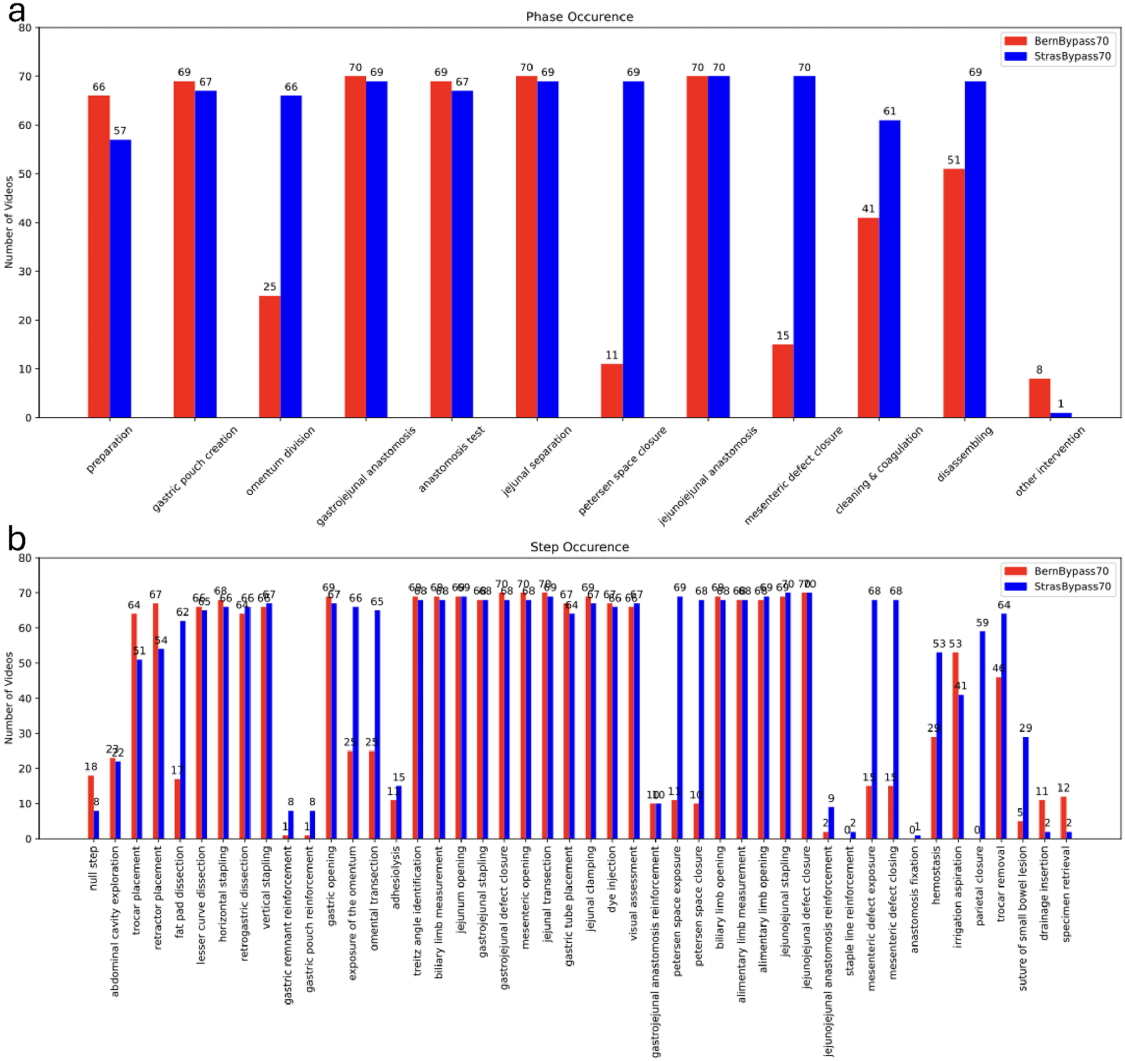
**a** Phase and **b** step occurrence in the MultiBypass140 dataset

MultiBypass140 included 140 LRYGB surgeries with a mean ± SD video duration of 92 ± 33 min. StrasBypass70 had a mean ± SD video duration of 111 ± 33 min and BernBypass70 of 73 ± 20 min. Phase occurrence differed significantly between StrasBypass70 and BernBypass70. Significant and systematic workflow variations due to different surgical techniques were observed between the StrasBypass70 and BernBypass70 datasets. Both the Petersen space (99% vs. 16%, *p* < 0.01) and the mesenteric defect (100% vs. 21%, *p* < 0.01) were systematically closed, while the greater omentum was routinely divided (94% vs. 36%, *p* < 0.01) in StrasBypass70 versus BernBypass70. Figure 1 shows the occurrence of phases and steps across the MultiBypass140 dataset.

A total of 797 iAEs were analyzed. Thereof, 658 (83%) were bleeding events, 117 (15%) were mechanical injuries, 12 (2%) were thermal injuries, 6 (1%) were ischemic injuries, and 4 (1%) were insufficient closures of anastomosis. On average 5.7 ± 3.2 events occurred per surgery. Baseline characteristics and the distribution of iAE type and grade in StrasBypass70 and BernBypass70 are shown in Table 2.

**Table 2:** Baseline characteristics and distribution of iAE type and grade in the dataset

	MultiBypass140	
	StrasBypass70	BernBypass70
Age (y, mean ± SD)	–	43.8 ± 13.9
Female sex (*n*, %)	–	59 (84.3)
BMI (kg/m^2^, mean ± SD)	–	39.7 ± 4.5
Video duration (min, mean ± SD)	111 ± 33	73 ± 20
Phases (*n*, mean)	10	8
Steps (*n*, mean)	33	27

Intraoperative adverse events (*n*, %)	Overall	Rectified	Overall	Rectified
Total	432 (100)	378 (87.5)	365 (100)	300 (82.2)
Bleeding	364 (84.3)	319 (73.8)	294 (80.5)	242 (66.3)
Grade 1	152 (35.2)	125 (28.9)	78 (21.4)	63 (17.3)
Grade 2	159 (36.8)	143 (33.1)	133 (36.4)	111 (30.4)
Grade 3	42 (9.7)	40 (9.3)	51 (14.0)	39 (10.7)
Grade 4	11 (2.5)	11 (2.5)	19 (5.2)	17 (4.7)
Grade 5	–	–	13 (3.6)	12 (3.2)
Mechanical injury	60 (13.9)	53 (12.3)	57 (15.6)	48 (13.2)
Grade 1	35 (8.1)	32 (7.4)	13 (3.6)	9 (2.5)
Grade 2	4 (0.9)	2 (0.5)	1 (0.3)	1 (0.3)
Grade 3	20 (4.6)	18 (4.2)	32 (8.8)	27 (7.4)
Grade 4	–	–	8 (2.2)	8 (2.2)
Grade 5	1 (0.2)	1 (0.2)	3 (0.8)	3 (0.8)
Thermal injury	6 (1.4)	4 (0.9)	6 (1.6)	5 (1.4)
Grade 1	2 (0.5)	1 (0.2)	–	–
Grade 2	–	–	–	–
Grade 3	4 (0.9)	3 (0.7)	5 (1.4)	4 (1.1)
Grade 4	–	–	1 (0.3)	1 (0.3)
Grade 5	–	–	–	–
Ischemic injury	1 (0.2)	1 (0.2)	5 (1.4)	2 (0.5)
Grade 3	1 (0.2)	1 (0.2)	4 (1.1)	2 (0.5)
Grade 5	–	–	1 (0.3)	–
Insufficient closure of the anastomosis	1 (0.2)	1 (0.2)	3 (0.8)	3 (0.8)

*SD* standard deviation

**Fig. 2:**
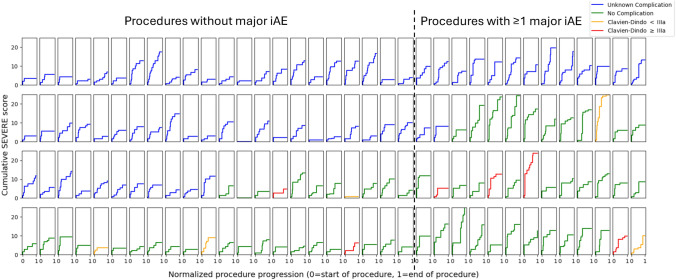
Progression of the cumulative intraoperative adverse event (iAE) severity (SEVERE score) per procedure in the MultiBypass140 dataset. Every panel represents one LRYGB procedure. The indexed procedure duration is shown on the x-axis (0 = start of the procedure; 1 = end of the procedure), whereas the cumulative severity of iAEs measured in SEVERE score is displayed on the y-axis. The dataset is stratified by occurrence of major iAE (left of the dashed line = procedures without major iAE; right of the dashed line = procedures with ≥ 1 major iAE) defined as 90th percentile contributing SEVERE score. Corresponding postoperative complications measured in Clavien–Dindo score are color coded: blue = not available; green = no complication; orange = grade < IIIa; and red = grade ≥ IIIa (Color figure online)

In total, 25% of procedures (35/140) had one major event and 12% of procedures (17/140) had more than one major event. The average number of minor events was not significantly different between procedures with and without major events (5.0 vs. 5.2, *p* = 0.76). The average number of minor events taking place before and after a major event did not differ significantly (2.4 vs. 2.7, *p* = 0.22). The average number of major events taking place before a major event was significantly lower than after a major event (0.8 vs. 1.3, *p* < 0.01). The progression of the SEVERE score over time stratified by procedures with and without major iAE is displayed in Fig. 2. Eighty-five percent (675/797) of iAEs were completely rectified.

Three phases of LRYGB surgery are the most iAE prone: gastric pouch creation (Phase 2, 293 iAEs, 37%), gastrojejunal anastomosis (Phase 4, 178 iAEs, 22%), and jejunojejunal anastomosis (Phase 8, 145 iAEs, 18%) (Table 3). Corresponding to the most iAE-prone phases, the following steps of LRYGB surgery are most iAE prone: lesser curve dissection (Step 5, 112 iAEs, 14%), gastrojejunal defect closure (Step 19, 118 iAEs, 15%), and jejunojejunal defect closure (Step 33, 85 iAEs, 11%).

**Table 3:** Frequencies and types of iAEs in the most eventful phases

		MultiBypass140	
Phase	iAE type	StrasBypass70	BernBypass70
Gastric pouch creation	Bleeding (*n*, %)	144 (94.1)	130 (93.5)
	Mechanical injury (*n*, %)	6 (3.9)	5 (3.6)
	Thermal injury (*n*, %)	3 (1.9)	2 (1.4)
	Ischemic injury (*n*, %)	–	2 (1.4)
	Insufficient closure of the anastomosis (*n*, %)	–	–
Gastrojejunal anastomosis	Bleeding (*n*, %)	64 (76.2)	77 (81.9)
	Mechanical injury (*n*, %)	20 (23.8)	13 (13.8)
	Thermal injury (*n*, %)	–	1 (1.1)
	Ischemic injury (*n*, %)	–	2 (2.1)
	Insufficient closure of the anastomosis (*n*, %)	–	1 (1.1)
Jejunojejunal anastomosis	Bleeding (*n*, %)	49 (68.1)	46 (63.0)
	Mechanical injury (*n*, %)	20 (27.8)	25 (34.2)
	Thermal injury (*n*, %)	3 (4.2)	2 (2.7)
	Ischemic injury (*n*, %)	–	–
	Insufficient closure of the anastomosis (*n*, %)	–	–

**Fig. 3:**
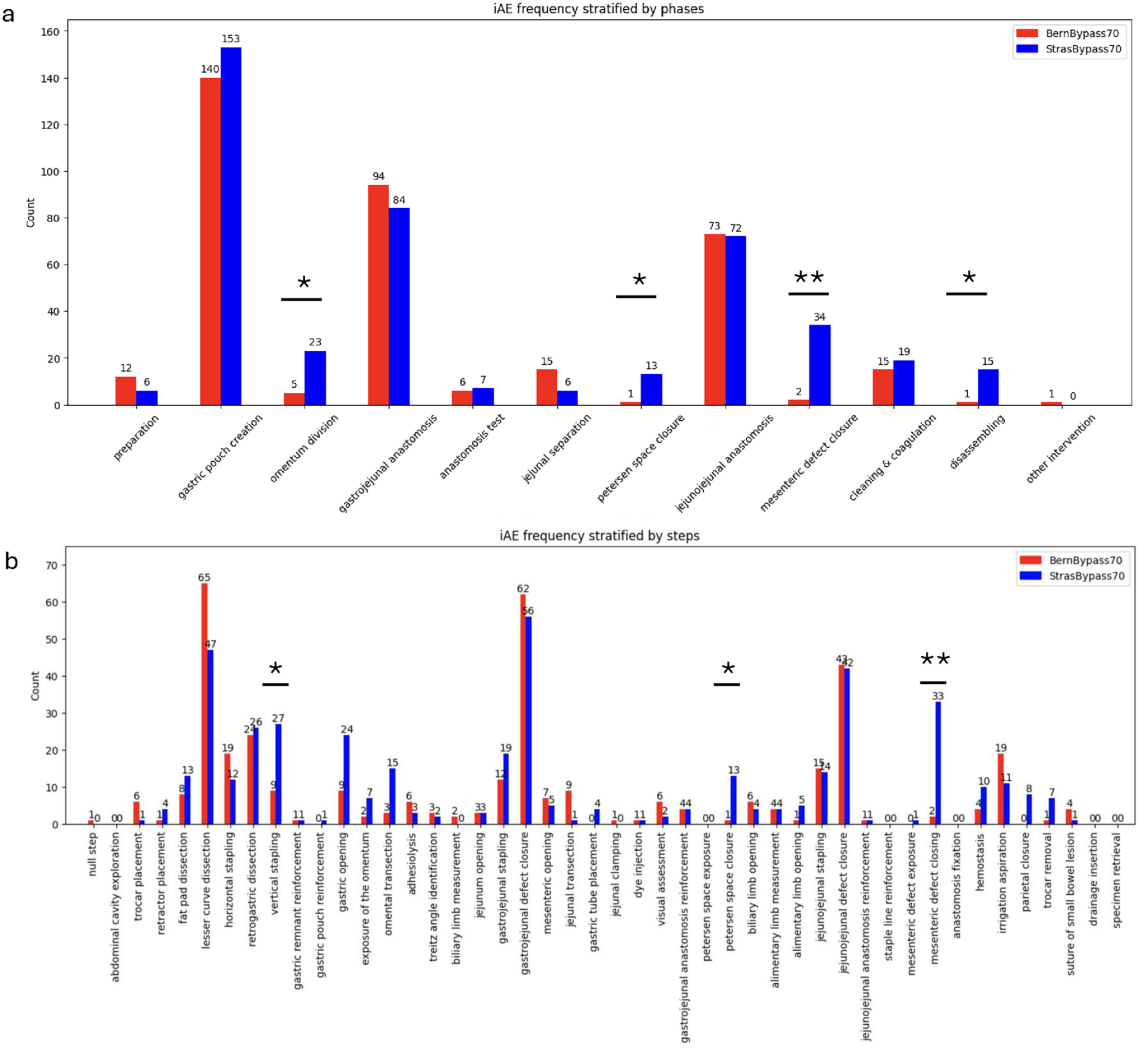
Intraoperative adverse event (iAE) frequency per **a** phase and **b** step stratified by center. *p ≤ 0.05, **p ≤ 0.01

The frequency of iAE occurrence was compared between centers. StrasBypass70 showed significantly more iAEs in the omentum division (Phase 3, 23 vs. 5 iAEs, *p* = 0.02), Petersen space closure (Phase 7, 13 vs. 1 iAEs, *p* = 0.03), mesenteric defect closure (Phase 9, 34 vs. 2 iAEs, *p* < 0.01), and disassembling phases (Phase 11, 15 vs. 1 iAEs, *p* = 0.02) when compared to BernBypass70 (Fig. 3a). The frequencies of iAE were also assessed on the step level. StrasBypass70 showed significantly more iAEs in the vertical stapling (Step 8, 27 vs. 9 iAEs, *p* = 0.05), Petersen space closure (Step 28, 13 vs. 1 iAEs, *p* = 0.03), and mesenteric defect closure (Step 37, 33 vs. 2 iAEs, *p* < 0.01) when compared to BernBypass70 (Fig. 3b).

**Fig. 4:**
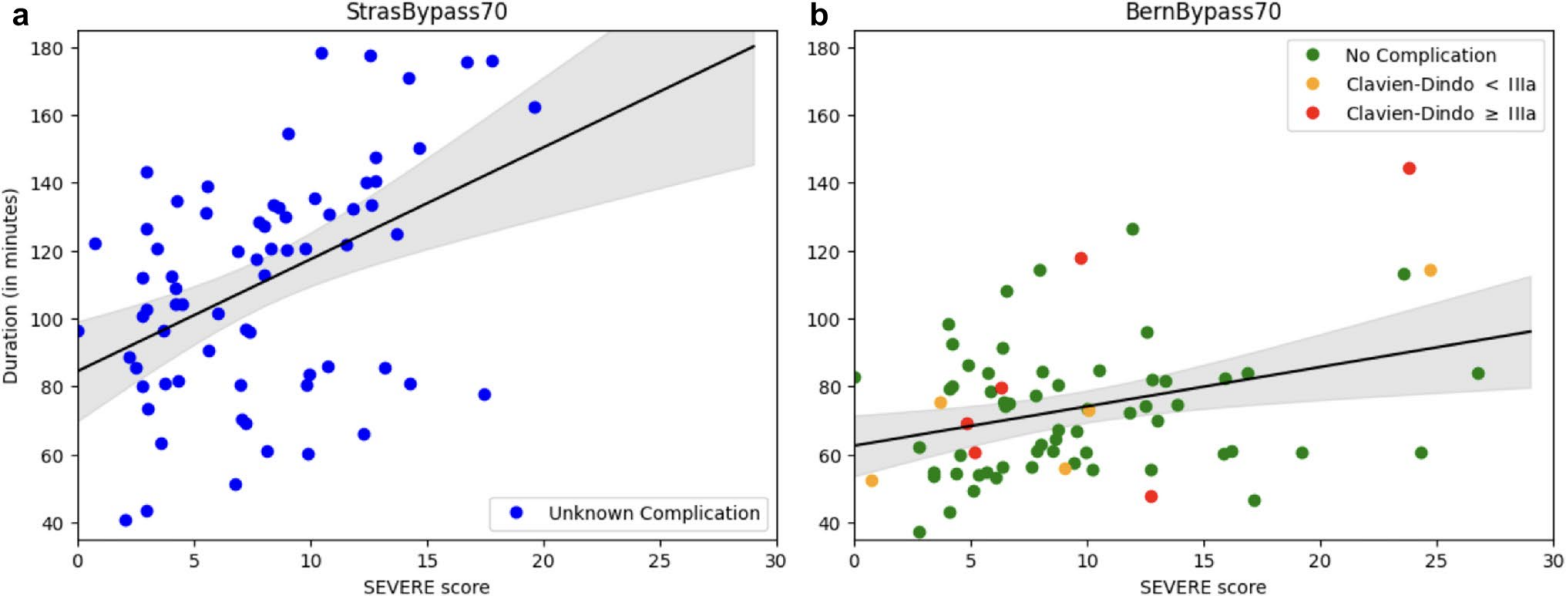
Correlation of SEVERE score and procedure duration for **a** StrasBypass70 (*r* = 0.44, *p* < 0.01) and **b** BernBypass70 (*r* = 0.33, *p* < 0.01). Regression lines with 95% confidence intervals

For both datasets, there was a positive correlation between SEVERE score and procedure duration (Fig. 4, StrassBypass70 *r* = 0.44, *p* < 0.01; BernBypass70 *r* = 0.33, *p* < 0.01). However, in BernBypass70, the operative time increased less per SEVERE score point than in StrasBypass70.

Out of 70 patients in BernBypass70, five patients (7%) underwent minor complications and six patients (9%) major postoperative complications. There was no significant difference in the average iAE frequency of patients with postoperative complications and those with an uncomplicated postoperative course (5.5 ± 2.5 vs. 5.1 ± 2.5 iAEs, *p* = 0.67). When stratifying according to iAE type, there were no significant differences in average bleeding events (4.3 ± 2.2 vs. 4.2 ± 2.1, *p* = 0.87), mechanical injuries (0.8 ± 1.0 vs. 0.8 ± 1.0, *p* = 1.00), thermal injuries (0.0 ± 0.0 vs. 0.1 ± 0.4, *p* = 0.27), and ischemic injuries (0.2 ± 0.4 vs. 0.1 ± 0.2, *p* = 0.26) between patients with postoperative complications and those with an uncomplicated postoperative course. Although these events were rare and all were rectified, insufficient closure of anastomosis was significantly more frequent in patients with postoperative complications (0.2 ± 0.6 vs. 0.0 ± 0.1, *p* < 0.01).

## Discussion

iAEs are important to study as they are often considered preventable [20, 21] and may contribute to the development of postoperative complications and influence outcomes. Previous work has shown that iAEs can be associated with increased procedure duration [13], which in turn could be associated with increased postoperative complications [9, 10]. Other work has suggested that minor events are often erroneously deemed inconsequential and may serve as a potential surrogate for performance assessment [22]. Still, the occurrence of iAEs is intricately intertwined with technical, organizational, human, and patient-related factors, limiting our understanding of the root cause and consequence of these events.

This study used video-based assessment to analyze the temporal occurrence, frequency, type, and grade of iAE in LRYGB surgery in a multicentric video dataset, aiming to shed light on the various links between surgical technique and iAEs. Several frameworks have been proposed to classify iAEs in surgery (Table 1) [23, 24]. Some classifications measure the cumulative severity of iAEs or the most severe single iAE assessing the intervention globally [25–27], other classifications like the SEVERE index assess multiple individual events during an intervention. Other classifications use postoperative outcomes measures like intensive care unit admission or reoperation to classify the severity of iAEs [28, 29]. Classifications relying on postoperative outcomes prohibit prospective applications, such as developing predictors of postoperative complications, as the downstream consequences of the observed iAE are not known beforehand. Only classifications relying solely on intraoperative data at the time point of iAE occurrence are suitable for prospective video-based assessment. Therefore, the SEVERE index was applied in this study.

A single-center study from Canada assessed iAE in 120 consecutive LRYGB surgeries with a median of 12 events per patient [13]. Despite using the same assessment tool (SEVERE index), the present study revealed on average 6 events per patient. Furthermore, the Canadian study does not give information on the temporal occurrence of iAE. Thus, with the inclusion of two high-volume academic centers along with corresponding phase and step information, the present study achieves a new level of detailed understanding of variations in surgical technique and the occurrence of iAEs. This fine-grained analysis of iAE in LRYGB enables data-driven feedback to trainees and researchers aiming to improve surgical technique.

The present study identified that gastric pouch, gastrojejunal anastomosis, and jejunojejunal anastomosis creation are the most iAE-prone phases (Fig. 3). In total, 77% of all iAEs in the MultiBypass140 dataset occur during those three phases. According to the ontology used, every phase has its corresponding steps. Therefore, the most iAE-prone steps correspond to the most iAE-prone phases. The most iAE-prone steps are lesser curve dissection, gastrojejunal defect closure, and jejunojejunal defect closure. Knowing the iAE-prone phases and steps of LRYGB facilitates case review for educational purposes, quality improvement programs, and mortality & morbidity conferences. Moreover, the knowledge of iAE-prone phases and steps enables safer teaching and focused video documentation [30] of LRYGB procedures. Surgical trainees can be closely mentored in iAE-prone phases of LRYGB. Further, to reduce iAEs and overcome the learning curve of LRYGB iAE-prone steps can be practiced in box model trainers.

Where in StrasBypass70 the greater omentum is usually divided and the mesenteric defects are systematically closed, this is not the case for most of the surgeries in BernBypass70. In a registry-based cohort study from Sweden routine division of the greater omentum in LRYGB has been shown to reduce postoperative small bowel obstruction [7]. However, omental division is associated with significantly increased overall iAEs and intraoperative bleeding. This finding can be confirmed by the present study. StrasBypass70 had significantly more iAEs in the omentum division phase and the omental transection step than BernBypass70.

Another difference in surgical technique between StrasBypass70 and BernBypass70 lies in the routine closure of the Petersen and the jejunal mesenteric defect in StrasBypass70. There is strong evidence that routine closure of mesenteric defects results in reduced incidence of small bowel obstruction and internal hernia [8, 31]. However, as the present study suggests this comes at the price of an increased frequency of iAEs in the respective phases and steps. The increased iAE frequency of StrasBypass70 compared to BernBypass70 is due to omentum division (Phase 3) and mesenteric defect closure (Phases 7 & 9). By omitting those phases, potentially 16% (70/432) of iAE could be prevented. These apparent trade-offs between improved complication rates despite increases in iAE rates further emphasize the need to study iAEs in conjunction with variations in technique and outcomes.

Irrespective of the differences in surgical technique, for both datasets, a correlation of the SEVERE score, which is the cumulative severity of iAEs per procedure, and the procedure duration has been observed (Fig. 4). The Multibypass140 dataset does not contain information regarding the technical skill level of the surgeons or the case difficulty. Nevertheless, the SEVERE score is likely a proxy for both. Examining the progression of SEVERE score over time, our study revealed that major iAE did not occur in isolation. Most major iAE have precedent or subsequent major or minor iAEs (Fig. 2). However, major events are not correlated with an increased frequency of minor events. Minor events are likely to take place before than after a major event. A major event increases the likelihood of subsequent major events. There is a large body of literature showing that prolonged operative duration is associated with postoperative complication [32]. However, it remains unknown whether increased surgery duration is the cause or the consequence of iAEs. As with procedure duration the number of iAEs and the cumulative iAE severity is increasing, iAEs might be one of the drivers for postoperative complications in patients with prolonged operative time. However, in BernBypass70, where postoperative outcomes were available, a correlation of iAE frequency and 30-day postoperative complications could only be shown for the most severe type of iAEs, which is insufficient closure of the anastomosis. iAEs may translate to postoperative complications but do not necessarily have to. Indeed, a vast majority of iAEs (65%) in this study were low-grade bleeding and completely rectified. They are unlikely to translate into postoperative complications. On the other hand, in this study, certain iAEs such as insufficient closure of anastomosis, though rare and always rectified, were significantly associated with postoperative complications. A need for granular, and potentially independent analysis, of various types of iAEs may be warranted. While iAEs may be predictors of postoperative complications, any robust tool will need to account for variations in surgical technique, operator skill, and patient characteristics, among other factors that could severely impact the occurrence of iAEs.

A strength of this study is its unprecedented granularity of iAE analysis. To our knowledge, there is no prior work analyzing iAEs with respect to the temporal occurrence and surgical technique. The present study is limited by the fact that the patients in StrasBypass70 and BernBypass70 are not matched. As StrasBypass70 is an anonymous dataset, no demographic or outcome variables are available and therefore patients cannot be matched with patients from BernBypass70. Thus, every comparison between the two datasets must be interpreted cautiously.

Future studies will need to include more centers with greater variability of surgical technique. To further enhance the understanding of the relationship of iAE with postoperative complications, the inclusion of more video datasets with corresponding outcome data is needed. The MultiBypass140 dataset and corresponding iAE annotations will serve as basis for the training of a deep learning iAE detection algorithm. This will lead to automated iAE detection in LRYGB and help to prevent the translation of iAE into postoperative complications.

In summary, this study used video-based assessment to study the frequency, the type, and grade of iAEs in LRYGB regarding surgical phases and steps. Gastric pouch creation, gastrojejunal anastomosis and jejunojejunal anastomosis are the most iAE-prone phases of LRYGB. Routine division of the greater omentum and closure of the mesenteric defects increases the frequency of iAEs. The cumulative severity of iAEs is correlated with operative time. An association of iAE with postoperative complications was solely shown for the most severe iAE.

## Supplementary Information

Below is the link to the electronic supplementary material.

Supplementary file1 (PDF 33 KB) Hierarchical structure of the laparoscopic Roux-en-Y gastric bypass phase (P) and step (S) ontology as proposed in [15]. Optional phases and steps have a dashed border

## References

[1] Gloy VL, Briel M, Bhatt DL, Kashyap SR, Schauer PR, Mingrone G, Bucher HC, Nordmann AJ (2013) Bariatric surgery versus non-surgical treatment for obesity: a systematic review and meta-analysis of randomised controlled trials. BMJ 347:f5934–f5934. 10.1136/bmj.f5934 24149519 10.1136/bmj.f5934 PMC3806364

[2] Colquitt JL, Pickett K, Loveman E, Frampton GK (2014) Surgery for weight loss in adults. Cochrane Database Syst Rev. 10.1002/14651858.CD003641.pub4 25105982 10.1002/14651858.CD003641.pub4 PMC9028049

[3] Welbourn R, Hollyman M, Kinsman R, Dixon J, Liem R, Ottosson J, Ramos A, Våge V, Al-Sabah S, Brown W, Cohen R, Walton P, Himpens J (2019) Bariatric surgery worldwide: baseline demographic description and one-year outcomes from the fourth IFSO global registry report 2018. Obes Surg 29:782–795. 10.1007/s11695-018-3593-1 30421326 10.1007/s11695-018-3593-1

[4] Brown W, Shikora S, Liem R, Holland J, Campbell AB, Sprinkhuisen SM, Kuijpers S, Kow L (2022) 7th IFSO global registry report. https://www.ifso.com/pdf/ifso-7th-registry-report-2022.pdf/ Accessed 15 May 2023

[5] Kaijser MA, van Ramshorst GH, Emous M, Veeger NJGM, van Wagensveld BA, Pierie J-PEN (2018) A Delphi consensus of the crucial steps in gastric bypass and sleeve gastrectomy procedures in the Netherlands. Obes Surg 28:2634–2643. 10.1007/s11695-018-3219-7 29633151 10.1007/s11695-018-3219-7 PMC6132743

[6] Gero D, Raptis DA, Vleeschouwers W, van Veldhuisen SL, Martin AS, Xiao Y, Galvao M, Giorgi M, Benois M, Espinoza F, Hollyman M, Lloyd A, Hosa H, Schmidt H, Garcia-Galocha JL, van de Vrande S, Chiappetta S, Menzo EL, Aboud CM, Lüthy SG, Orchard P, Rothe S, Prager G, Pournaras DJ, Cohen R, Rosenthal R, Weiner R, Himpens J, Torres A, Higa K, Welbourn R, Berry M, Boza C, Iannelli A, Vithiananthan S, Ramos A, Olbers T, Sepúlveda M, Hazebroek EJ, Dillemans B, Staiger RD, Puhan MA, Peterli R, Bueter M (2019) Defining global benchmarks in bariatric surgery: a retrospective multicenter analysis of minimally invasive Roux-en-Y gastric bypass and sleeve gastrectomy. Ann Surg 270:859–867. 10.1097/SLA.0000000000003512 31592894 10.1097/SLA.0000000000003512

[7] Josefsson E, Ottosson J, Näslund I, Näslund E, Stenberg E (2023) The effect of routine division of the greater omentum on small bowel obstruction after Roux-en-Y gastric bypass. Surg Obes Relat Dis 19:178–183. 10.1016/j.soard.2022.09.006 36207233 10.1016/j.soard.2022.09.006

[8] Stenberg E, Szabo E, Ågren G, Ottosson J, Marsk R, Lönroth H, Boman L, Magnuson A, Thorell A, Näslund I (2016) Closure of mesenteric defects in laparoscopic gastric bypass: a multicentre, randomised, parallel, open-label trial. The Lancet 387:1397–1404. 10.1016/S0140-6736(15)01126-5 10.1016/S0140-6736(15)01126-5 26895675

[9] Stenberg E, Szabo E, Ågren G, Näslund E, Boman L, Bylund A, Hedenbro J, Laurenius A, Lundegårdh G, Lönroth H, Möller P, Sundbom M, Ottosson J, Näslund I (2014) Early complications after laparoscopic gastric bypass surgery: results from the scandinavian obesity surgery registry. Ann Surg 260:1040–1047. 10.1097/SLA.0000000000000431 24374541 10.1097/SLA.0000000000000431

[10] Bohnen JD, Mavros MN, Ramly EP, Chang Y, Yeh DD, Lee J, De Moya M, King DR, Fagenholz PJ, Butler K, Velmahos GC, Kaafarani HMA (2017) Intraoperative adverse events in abdominal surgery: what happens in the operating room does not stay in the operating room. Ann Surg 265:1119–1125. 10.1097/SLA.0000000000001906 27805961 10.1097/SLA.0000000000001906

[11] Mavros MN, Bohnen JD, Ramly EP, Velmahos GC, Yeh DD, De Moya M, Fagenholz P, King DR, Lee J, Kaafarani HMA (2015) Intraoperative adverse events: risk adjustment for procedure complexity and presence of adhesions is crucial. J Am Coll Surg 221:345–353. 10.1016/j.jamcollsurg.2015.03.045 26141463 10.1016/j.jamcollsurg.2015.03.045

[12] Pugh CM, Hashimoto DA, Korndorffer JR (2021) The what? How? And Who? Of video based assessment. Am J Surg 221:13–18. 10.1016/j.amjsurg.2020.06.027 32665080 10.1016/j.amjsurg.2020.06.027

[13] Jung JJ, Jüni P, Gee DW, Zak Y, Cheverie J, Yoo JS, Morton JM, Grantcharov T (2020) Development and evaluation of a novel instrument to measure severity of intraoperative events using video data. Ann Surg 272:220–226. 10.1097/SLA.0000000000003897 32675485 10.1097/SLA.0000000000003897

[14] Lavanchy JL, Ramesh S, Dall’Alba D, Gonzalez C, Fiorini P, Müller-Stich BP, Nett PC, Marescaux J, Mutter D, Padoy N (2024) Challenges in multi-centric generalization: phase and step recognition in Roux-en-Y gastric bypass surgery. Int J Comput Assist Radiol Surg. 10.1007/s11548-024-03166-3 10.1007/s11548-024-03166-3 PMC11541311 38761319

[15] Lavanchy JL, Gonzalez C, Kassem H, Nett PC, Mutter D, Padoy N (2022) Proposal and multicentric validation of a laparoscopic Roux-en-Y gastric bypass surgery ontology. Surg Endosc 37:2070–2077. 10.1007/s00464-022-09745-2 36289088 10.1007/s00464-022-09745-2 PMC10017621

[16] Mazellier J-P, Boujon A, Bour-Lang M, Erharhd M, Waechter J, Wernert E, Mascagni P, Padoy N (2023) MOSaiC: a web-based platform for collaborative medical video assessment and annotation. Preprint at https://arxiv.org/abs/2312.08593

[17] Dindo D, Demartines N, Clavien PA (2004) Classification of surgical complications: a new proposal with evaluation in a cohort of 6336 patients and results of a survey. Ann Surg 240:205–213. 10.1097/01.sla.0000133083.54934.ae 15273542 10.1097/01.sla.0000133083.54934.ae PMC1360123

[18] Von Elm E, Altman DG, Egger M, Pocock SJ, Gøtzsche PC, Vandenbroucke JP (2007) The strengthening the reporting of observational studies in epidemiology (STROBE) statement: guidelines for reporting observational studies. The Lancet 370:1453–1457. 10.1016/S0140-6736(07)61602-X 10.1016/S0140-6736(07)61602-X 18064739

[19] Lavanchy JL, Vardazaryan A, Mascagni P, Consortium A, Mutter D, Padoy N (2023) Preserving privacy in surgical video analysis using a deep learning classifier to identify out-of-body scenes in endoscopic videos. Sci Rep 13:9235. 10.1038/s41598-023-36453-1 37286660 10.1038/s41598-023-36453-1 PMC10247775

[20] Zegers M, Hesselink G, Geense W, Vincent C, Wollersheim H (2016) Evidence-based interventions to reduce adverse events in hospitals: a systematic review of systematic reviews. BMJ Open 6:e012555. 10.1136/bmjopen-2016-012555 27687901 10.1136/bmjopen-2016-012555 PMC5051502

[21] Howell A-M, Panesar SS, Burns EM, Donaldson LJ, Darzi A (2014) Reducing the burden of surgical harm: a systematic review of the interventions used to reduce adverse events in surgery. Ann Surg 259:630–641. 10.1097/SLA.0000000000000371 24368639 10.1097/SLA.0000000000000371

[22] Curtis NJ, Dennison G, Brown CSB, Hewett PJ, Hanna GB, Stevenson ARL, Francis NK (2021) Clinical evaluation of intraoperative near misses in laparoscopic rectal cancer surgery. Ann Surg 273:778–784. 10.1097/SLA.0000000000003452 31274657 10.1097/SLA.0000000000003452

[23] Cacciamani GE, Sholklapper T, Dell’Oglio P, Rocco B, Annino F, Antonelli A, Amenta M, Borghesi M, Bove P, Bozzini G, Cafarelli A, Celia A, Leonardo C, Ceruti C, Cindolo L, Crivellaro S, Dalpiaz O, Falabella R, Falsaperla M, Galfano A, Gallo F, Greco F, Minervini A, Parma P, Chiara Sighinolfi M, Pastore AL, Pini G, Porreca A, Pucci L, Sciorio C, Schiavina R, Umari P, Varca V, Veneziano D, Verze P, Volpe A, Zaramella S, Lebastchi A, Abreu A, Mitropoulos D, Shekhar Biyani C, Sotelo R, Desai M, Artibani W, Gill I (2022) The Intraoperative complications assessment and reporting with universal standards (ICARUS) global surgical collaboration project: development of criteria for reporting adverse events during surgical procedures and evaluating their impact on the postoperative course. Eur Urol Focus 8:1847–1858. 10.1016/j.euf.2022.01.018 35177353 10.1016/j.euf.2022.01.018

[24] Sayegh AS, Eppler M, Sholklapper T, Goldenberg MG, Perez LC, La Riva A, Medina LG, Sotelo R, Desai MM, Gill I, Jung JJ, Kazaryan AM, Edwin B, Biyani CS, Francis N, Kaafarani HM, Cacciamani GE (2023) Severity grading systems for intraoperative adverse events. a systematic review of the literature and citation analysis. Ann Surg 278:e973–e980. 10.1097/SLA.0000000000005883 37185890 10.1097/SLA.0000000000005883

[25] Tang B, Hanna GB, Joice P, ChB M, Cuschieri A (2004) Identification and categorization of technical errors by observational clinical human reliability assessment (OCHRA) during laparoscopic cholecystectomy. Arch Surg 139:1215–1220. 10.1001/archsurg.139.11.1215 15545569 10.1001/archsurg.139.11.1215

[26] Rosenthal R, Hoffmann H, Clavien PA, Bucher HC, Dell-Kuster S (2015) Definition and classification of intraoperative complications (classic): Delphi study and pilot evaluation. World J Surg 39:1663–1671. 10.1007/s00268-015-3003-y 25665678 10.1007/s00268-015-3003-y

[27] Dell-Kuster S, Gomes NV, Gawria L, Aghlmandi S, Aduse-Poku M, Bissett I, Blanc C, Brandt C, Ten Broek RB, Bruppacher HR, Clancy C, Delrio P, Espin E, Galanos-Demiris K, Gecim IE, Ghaffari S, Gié O, Goebel B, Hahnloser D, Herbst F, Orestis I, Joller S, Kang S, Martín R, Mayr J, Meier S, Murugesan J, Nally D, Ozcelik M, Pace U, Passeri M, Rabanser S, Ranter B, Rega D, Ridgway PF, Rosman C, Schmid R, Schumacher P, Solis-Pena A, Villarino L, Vrochides D, Engel A, O’grady G, Loveday B, Steiner LA, Van Goor H, Bucher HC, Clavien PA, Kirchhoff P, Rosenthal R, (2020) Prospective validation of classification of intraoperative adverse events (ClassIntra): international, multicentre cohort study. The BMJ 370:m2917. 10.1136/bmj.m2917 32843333 10.1136/bmj.m2917 PMC7500355

[28] Kaafarani HMA, Mavros MN, Hwabejire J, Fagenholz P, Yeh DD, Demoya M, King DR, Alam HB, Chang Y, Hutter M, Antonelli D, Gervasini A, Velmahos GC (2014) Derivation and validation of a novel severity classification for intraoperative adverse events. J Am Coll Surg 218:1120–1128. 10.1016/j.jamcollsurg.2013.12.060 24702887 10.1016/j.jamcollsurg.2013.12.060

[29] Francis NK, Curtis NJ, Conti JA, Foster JD, Bonjer HJ, Hanna GB, Abu-Hilal M, Agresta F, Antoniu SA, Arezzo A, Balagúe C, Boni L, Bouvy N, Carus T, Edwin B, Diana M, Faria G, Ignjatovic D, de Manzini N, Margallo FM, Martinek L, Matveev N, Mintz Y, Nakajima K, Popa DE, Schijven PJ, Sedman P, Yiannakopoulou E (2018) EAES classification of intraoperative adverse events in laparoscopic surgery. Surg Endosc 32:3822–3829. 10.1007/s00464-018-6108-1 29435754 10.1007/s00464-018-6108-1

[30] Mascagni P, Alapatt D, Urade T, Vardazaryan A, Mutter D, Marescaux J, Costamagna G, Dallemagne B, Padoy N (2021) A computer vision platform to automatically locate critical events in surgical videos: documenting safety in laparoscopic cholecystectomy. Ann Surg 274:e93–e95. 10.1097/SLA.0000000000004736 33417329 10.1097/SLA.0000000000004736

[31] Magouliotis DE, Tzovaras G, Tasiopoulou VS, Christodoulidis G, Zacharoulis D (2020) Closure of mesenteric defects in laparoscopic gastric bypass: a meta-analysis. Obes Surg 30:1935–1943. 10.1007/s11695-020-04418-2 31955371 10.1007/s11695-020-04418-2

[32] Cheng H, Clymer JW, Po-Han Chen B, Sadeghirad B, Ferko NC, Cameron CG, Hinoul P (2018) Prolonged operative duration is associated with complications: a systematic review and meta-analysis. J Surg Res 229:134–144. 10.1016/j.jss.2018.03.022 29936980 10.1016/j.jss.2018.03.022

