# Supplementary material for: Analyzing the impact of surgical technique on intraoperative adverse events in laparoscopic Roux-en-Y gastric bypass surgery by video-based assessment
Source: Surg Endosc. 2025 Jan 31;39(3):2026–36. doi: 10.1007/s00464-025-11557-z (PMC11870895; doi:10.1007/s00464-025-11557-z)
Supplement: Supplementary file 1 — Supplementary file1 (PDF 33 KB) Hierarchical structure of the laparoscopic Roux-en-Y gastric bypass phase (P) and step (S) ontology as proposed in [15]. Optional phases and steps have a dashed border [file 464_2025_11557_MOESM1_ESM.pdf]

Phase	Steps
P1 preparation	S0 null step; S1 abdominal cavity exploration; S2 trocar placement; S3 retractor placement; S14 adhesiolysis; S22 gastric tube placement; S39 hemostasis; S40 irrigation aspiration
P2 gastric pouch creation	S0 null step; S4 fat pad dissection; S5 lesser curve dissection; S6 horizontal stapling; S7 retrogastric dissection; S8 Vertical stapling; S9 gastric remnant reinforcement; S10 gastric pouch reinforcement; S11 gastric opening; S22 gastric tube placement; S39 hemostasis; S40 irrigation aspiration
P3 omentum division	S0 null step; S12 omentum exposure; S13 omental transection; S39 hemostasis
P4 gastrojejunal anastomosis	S0 null step; S15 Treitz angle identification; S16 biliary limb measurement; S17 jejunum opening; S18 gastrojejunal stapling; S19 gastrojejunal defect closure; S26 gastrojejunal anastomosis reinforcement; S39 hemostasis; S40 irrigation aspiration
P5 anastomosis test	S0 null step; S3 retractor placement; S22 gastric tube placement; S23 jejunal clamping; S24 dye injection; S25 visual assessment; S39 hemostasis; S40 irrigation aspiration
P6 jejunal separation	S0 null step; S3 retractor placement; S20 mesenteric opening; S21 jejunal transection; S39 hemostasis; S40 irrigation aspiration
P7 Petersen space closure	S0 null step; S27 Petersen space exposure; S28 Petersen space closure
P8 Jejunojejunal anastomosis	S0 null step; S29 biliary limb opening; S30 alimentary limb measurement; S31 alimentary limb opening; S32 jejunojejunal stapling; S33 jejunojejunal defect closure; S34 jejunojejunal anastomosis reinforcement; S35 staple line reinforcement; S39 hemostasis; S40 irrigation aspiration
P9 Mesenteric defect closure	S0 null step; S36 mesenteric defect exposure; S37 mesenteric defect closure; S38 anastomosis fixation
P10 Cleaning & Coagulation	S0 null step; S39 hemostasis; S40 irrigation aspiration; S43 suture of small bowel lesion
P11 Disassembling	S0 null step; S41 parietal closure; S42 trocar removal; S44 drainage insertion; S45 specimen retrieval
P12 Other intervention	
